# Subgroups of relational job characteristics and their differences in turnover intention and subjective well-being among nurses: a latent profile analysis

**DOI:** 10.1186/s12912-024-02141-2

**Published:** 2024-07-08

**Authors:** Yi-ping Chen, Yifei Li, Jie Zhang, Juan Li, Xiu-fen Yang, Lile Xiong, Guili Xia, Jingping Zhang

**Affiliations:** 1 Department of Gastroenterology, Shenzhen Hospital, Southern Medical University, Shenzhen, Guangdong China; 2 Xiangya Nursing School of Central South University, Changsha, Hunan China; 3 Hunan University of Chinese Medicine, Changsha, Hunan China; 4 Shenzhen People’s Hospital, Shenzhen, Guangdong China; 5 Shenzhen Clinical Research Center for Digestive Disease, Shenzhen, China

**Keywords:** Clinical nurse, Relational job characteristics, Turnover intention, Subjective well-being, Latent profile analysis

## Abstract

**Objective:**

Relational job characteristics include perceived social worth and perceived social influence. Good relational job characteristics mean that nurses have high prosocial behavior. The purpose of this study was to explore the potential profile of nurses’ relational job characteristics, influencing factors and their differences in turnover intention and subjective well-being, thus finding the most suitable clinical relationship job characteristics.

**Methods:**

A cross-sectional survey was conducted among 1013 clinical nurses using the general demographic data questionnaire, Relational Job Characteristics scale, Turnover Intention Questionnaire and Campbell index of well-being. A latent profile analysis was performed to explore relational job characteristics latent profiles. Multinomial logistic regression analysis was conducted to examine the predictors of profile membership, and a one-way analysis of variance was applied to compare the turnover intention and subjective well-being in each latent profile.

**Results:**

Five latent profiles were identified and labeled ‘High prosocial job characteristics’ profile (20.7%), ‘Moderate prosocial job characteristics’ profile (41.7%), ‘High social worth-low social impact perceived’ profile (6.3%), ‘Low social worth‐high social impact perceived’ profile (18.8%) and ‘Low prosocial job characteristics’ profile (12.5%). Factors affecting the different types of nurse relationship job characteristics include age, marital status, hospital department, nursing years, professional title and hospital position. Among them, chief nurse, nurses with more than 20 years of nursing experience and obstetrics and gynecology nurses were more likely to be ‘high prosocial job characteristics’ profile. The turnover intention of nurses in ‘high prosocial job characteristics’ profile was significantly lower than that of other profiles, and their subjective well-being was significantly higher than that of other profiles.

**Conclusion:**

Improving nurses’ perception of social worth and social impact on clinical work can improve nurses’ prosocial behavior and subjective well-being, and reduce their turnover intention. Nursing managers or policy makers can formulate targeted intervention measures according to the influencing factors of potential profiles.

## Introduction

The shortage of nurses has become a serious problem, with a negative impact on global healthcare [1]. In the United Kingdom, there are over 41 000 vacant nursing posts across the United Kingdom’s National Health Service, with more people leaving the profession that joining it [2]. In China, the shortage of nurses is so serious that there are only 3.71 registered nurses per 1,000 people [3], which is still far from the minimum standard of 4.45 nurses per 1,000 people recommended by the World Health Organization [4]. And the World Health Organization estimates that the shortage of skilled health professionals will reach 12.9 million in 2035 [2].

Turnover is the most recognized reason for nursing shortage. Nursing turnover not only affect the quality of clinical care and pose a threat to patient safety, but can also increase hospital recruitment and training costs [5, 6]. Turnover intention, a willingness of employees to voluntarily leave a particular organization during the work period [7], can serve as the most effective predictor of the actual turnover behavior among nurses [8]. Meanwhile, as an important indicator to measure the quality of personal and social life, the studies found that subjective well-being can effectively improve nurses’ work enthusiasm and job satisfaction [9, 10], and is also an important predictor of nurses’ turnover intention and actual turnover behavior [11]. Therefore, how to improve the subjective well-being of nurses and reduce their turnover intention from the perspective of positive psychology has become the focus of current nursing managers.

Relational job characteristics are defined as the contact professionals have with clients and the impact on client lives [12]. High relational job characteristics mean that nurses have higher prosocial attributes [13]. Prosocial behavior, also known as prosocial service behavior, refers to a kind of behavior in which the actor voluntarily brings benefits to the recipient of the behavior [14]. As a helping profession whose main task is to provide medical services [15], the altruism and prosocial behavior that want to help and benefit others is one of the important qualities that every medical staff should have [16, 17]. Numerous studies have shown that prosocial behavior can be used as one of the positive work indicators of nurses, such as nurses with high prosocial behavior tend to have higher subjective well-being [18], job engagement [19], retention intention [20], and lower perceived burnout [21]. Furthermore, research has revealed associations of relational job characteristics with positive organizational and work outcomes [22]. Dai’s [23] research shows that nurses with high relational job characteristics tend to have better nurse-patient communication skills and nursing service quality. At the organizational level, relational job characteristics can also help improve team performance and team assistance, and create a harmonious and trusting working environment [24]. Therefore, as an important factor that can benefit hospitals, nurses and patients at the same time, and can predict nurses’ turnover intention and subjective well-being, how to improve nurses’ ability of relational job characteristics has become the focus of nurses and nursing students training.

Although many studies have explored relational job characteristics of nurses, they have mainly adopted variable-centered analysis methods, such as exploring the relationship between nurses’ relational job characteristics and their subjective well-being and job engagement, which may ignore individual heterogeneity [18, 25]. However, investigating the different patterns of relational job characteristics and tailoring interventions are both critical, as it enables nursing administrators and educators to help and support nurses or nursing students with different patterns of relational job characteristics in a targeted and ensure their fitness for future nursing work. To date, studies that have examined the relational job characteristics of nurses across different countries neither indicated a cut-off for distinguishing different levels nor provided a relevant reference [22, 26, 27]. Moreover, relational job characteristics include perceived social worth and perceived social impact subscales. In this case, evaluating the average score is generally too simple and cannot differentiate between subgroups of nurses with different patterns of relational job characteristics, and a ‘person-centered’ approach is more appropriate [28].

Latent profile analysis (LPA) is a person-centered algorithm that moves away from exploring the interrelationships between variables and treats variables as an interdependent system [29]. LPA can classify individuals according to data, capture group heterogeneity, and objectively identify different types of nurses’ relational job characteristics, which is of great significance for improving nurses’ relational job characteristics and formulating targeted intervention strategies for each profile. Therefore, exploring different patterns of nurse relational job characteristics using LPA and comparing the turnover intention and subjective well-being of nurses with different patterns of relational job characteristics may better clarify the beneficial impact of relational job characteristics on medical institutions, patients and nurses. It also helps us to find the most suitable patterns of relational job characteristics for nursing work, so as to help nursing managers and educators to develop and carry out follow-up cultivation plan.

This study employed LPA to (a) explore potentially different profiles in relational job characteristics, (b) identify the characteristics of each profile, and (c) compare the turnover intention and subjective well-being of different latent profiles, thus finding the most suitable clinical relationship job characteristics, to provide targeted guidance for nurses and nursing students intervention training, in order to help medical institutions, nurses and patients benefit.

## Methods

### Design

This study was a cross-sectional study conducted from 8 November 2021 to 10 March 2022 in five hospitals in the provinces of Hunan, Henan, and Guangdong, China.

### Participants

Nurses at five hospitals in China, were recruited as the research participants. The eligibility criteria included: (1) the hospital is a comprehensive hospital, (2) nurses have been registered and are on duty, (3) nurses are currently engaged in clinical practice, and (4) nurses are willing to participate in the study. Nurses who are interns, studying in other hospitals, or who have participated in other relevant studies were excluded from the study.

### Sample size

Previous studies have confirmed that the minimum sample size of LPA is 500 [30]. A total of 1013 participants were included in this study, which met the aforementioned sample size requirements.

### Data collection

Convenience sampling method was adopted in this study, and electronic questionnaire links were generated through the online data collection website “Wen Juanxing” (https://www.wjx.cn/) from 8 November 2021 to 10 March 2022. Data is collected anonymously through targeted, snowballing methods. Participants who meet the inclusion criteria can open the link using their mobile phone or computer, and an electronic informed consent will appear. If participants agree to accept the survey, they can enter the questionnaire survey interface to fill in the questionnaire. A pilot testing of 30 clinical nurses conducted in October 2021 showed that 200s was the minimum time to complete the questionnaire. In the formal surveys, questionnaires that are answered in less than 200s to complete will be deleted.

### Instruments

#### Demographic and job-related characteristics

A self-compiled online questionnaire was used to collect the individual characteristics of the latent profiles of relational job characteristics, including both demographic data (gender, age, education level, marital status and whether have any children) and work-related information (hospital level, hospital department, years of nursing experience, professional title, hospital position and employment type).

#### Relational job characteristics scale

The Relational Job Characteristics Scale was used to evaluate the level of prosocial job characteristics. The original version was developed by Grant [12] and then translated into Chinese by Chen [31], which was authorized by Grant. The scale has 18 items with two subscales: perceived social worth and perceived social impact. A seven-point Likert scale was used (1 = “disagree strongly” to 7 = “agree strongly”). Higher scores indicated higher levels of relational job characteristics. The reliability of the Relational Job Characteristics Scale among Chinese nurses was 0.964 and the Cronbach’s alpha of the scale in this study was 0.976.

#### Turnover intention questionnaire

The Turnover Intention Questionnaire aimed to measure nurses’ turnover intention. Michael [32] developed the original version. Li [33] translated it into Chinese after obtaining authorization from the original developers. The scale has 6 items with three subscales: the possibility of quitting your current job (1, 6), motivation to find other jobs (2, 3), and the possibility of getting outside work (4, 5). It is a 4-point scale (1–4, representing from ‘never’ to ‘always’) to measures turnover intention. Higher scores indicated higher levels of turnover intention. Cronbach’s alpha for the scale was 0.793.

#### Campbell index of well-being

The Chinese version of the Campbell Index of Well-being is widely used to measure the subjective well-being of Chinese people [34, 35], with two subsections: index of general affect, and overall life satisfaction item. The eight-item index of general affect asks participants to rate how often they experience a variety of emotions on a scale from 1 (very dissatisfied) to 7 (very satisfied). The overall life satisfaction item is composed of a single item that asks “How satisfied are you with your life as a whole?” and scored on a 7-point Likert scale (1 = very dissatisfied, 7 = very satisfied). Index of Well-Being = 1.1 * (overall life satisfaction item) + 1.0 * (Index of General Affect). Cronbach’s alpha for the scale in this study was 0.948.

#### Data analysis

SPSS26.0 software was used for statistical description, logistic regression analysis, one-way analysis of variance and the Least-Significant-Difference (LSD) test. Mplus8.3 software was used for latent profile analysis. The adaptation test indexes of potential profile model included: (1) Akaike Information Criterion (AIC), Bayesian Information Criterion (BIC) and Adjusted Bayesian Information Criterion (aBIC), the smaller the values of the three indicators, the better the fit of the model [29]. (2) Entropy represents the accuracy of classification, the value range is 0 ~ 1, the higher the Entropy value, the higher the accuracy of the classification [29]. (3) The P-value was tested based on Lo-Mendell Rubin Likelihood Test (LMR) and Bootstrap Likelihood Ratio Test (BLRT). *P* < 0.05 indicates that the model is significantly better than the previous model [36]. In this study, the results of each model are comprehensively judged to determine the best model. Logistic regression analyses were used to assess the effect of demographic and job-related characteristics on the relational job characteristics of different profiles of nurses. One-way analysis of variance and LSD were used to determine the subjective well-being and turnover intention among nurses with different profiles of relational job characteristics.

## Results

A total of 1058 electronic questionnaires were issued, and 1013 questionnaires were returned, for a response rate of 95.7%. The mean age of the 1013 participants was 31.8 (standard deviation [SD] = 7.2) years (ranging from 20 to 58). Most of the nurses were female (97.0%). More than two thirds of the participants had a bachelor’s degree (66.9%). Approximately 67.6% of the participants were married, and nearly two thirds of the participants had children (63.8%). Nearly three-quarters of the participants worked in third-class hospitals (74.8%). More than a third of the participants worked in internal medicine (35.8%). Nearly a third of the participants had been working in the nursing profession for 11 to 20 years (35.8%). More than a third of the participants had the title of ‘senior registered nurse’ (35.6%) and ‘supervisor nurse’ (34.3%). Over half of the participants had the position of ‘nurse’ (68.3%). Additionally, nearly three‐quarters of the participants were contract employed nurse (71.4%).

### Latent profiles of relational job characteristics

Six models were estimated during exploration, whose fit metrics are shown in Table 1. The Log(L), AIC, BIC, and aBIC values in the five-profile model were lower than those of the four-profile model, and the entropy value was higher than 0.9. The LMR value (*p* = 0.767) of the six-profile model was nonsignificant, which indicates that the five‐profile model is better than the six‐profile model. Overall, the five-profile model was optimal, and the fit metrics are highlighted in bold in Table 1.

**Table 1:** Fit metrics of each model

Model	k	Log(L)	AIC	BIC	aBIC	Entropy	LMR	BLRT
1 profile	36	−28397.618	56867.236	57044.381	56930.042	–	–	–
2 profiles	55	−23774.853	47659.706	47930.343	47755.658	0.962	0.0000	0.0000
3 profiles	74	−22254.837	44657.673	45021.803	44786.773	0.963	0.0012	0.0000
4 profiles	93	−21449.037	43084.074	43541.697	43246.321	0.954	0.0390	0.0000
**5 profiles**	**112**	−**20596.346**	**41416.693**	**41967.808**	**41612.087**	**0.965**	**0.0400**	**0.0000**
6 profiles	131	−20257.312	40776.624	41421.232	41005.166	0.966	0.7668	0.0000

*Abbreviations* k, Number of free parameters; Log(L), Log-likelihood value; AIC, Akaike information criterion; BIC, Bayesian information criteria; aBIC, adjusted Bayesian information criteria; LMR, Lo–Mendell–Rubin Test; BLRT, Bootstrap Likelihood Ratio Test

**Fig. 1:**
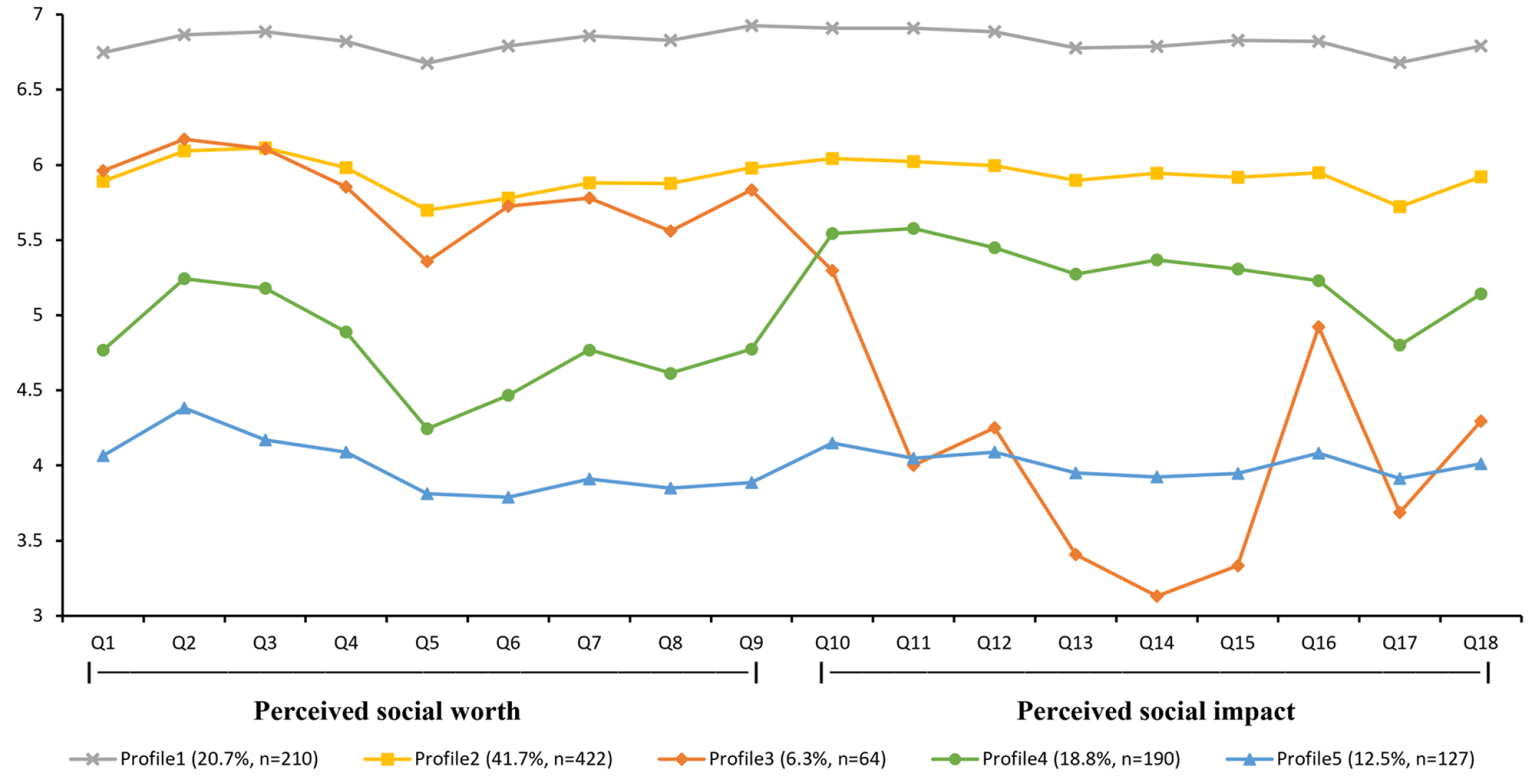
Latent profiles of relational job characteristics among nurses

The scores of five profiles on 18 items of two dimensions are shown in Fig. 1. Profile 1 was named the ‘high prosocial job characteristics’ group, accounting for 20.7% (*n* = 210) of all participants. It was notable that nurses in this profile reported the highest score for all items. Nurses in Profile 2 showed a moderate level of all items, which accounted for 41.7% (*n* = 422) of the sample. Therefore, this subgroup was named ‘moderate prosocial job characteristics ’. For nurses in Profile 3, their response rates of ‘agree’ to half of the items in the ‘perceived social worth’ dimension were much higher than average, whilst their responses were ‘disagree’ to most of the items in the ‘perceived social impact’ dimensions. Therefore, this subgroup was named the ‘high social worth-low social impact perceived’ group and accounted for 6.3% (*n* = 64). Profile 4 was named the ‘low social worth‐high social impact perceived’ group and accounted for 18.8% (*n* = 190). The average scores of ‘perceived social impact’ dimension were higher than that for ‘perceived social worth’ dimension in Profile 4. Profile 5 was named the ‘low prosocial job characteristics’ group because it had the lowest scores on the most items. Profile 5 accounted for 12.5% (n = 127) of the sample.

### Demographic and job-related characteristics of each profile

The demographic and job-related characteristics of the participants are presented in Table 2. The ‘high prosocial job characteristics’ group accounted for the largest percentage of nurses who married (72.9% vs. 69.7% vs. 64.1% vs. 65.3% vs. 57.5%) and had children (70.0% vs. 65.4% vs. 59.4% vs. 62.1% vs. 52.8%). The ‘low prosocial job characteristics’ group accounted for the smallest proportion of nurses who had a associate degree and below (39.4% vs. 26.3% vs. 23.4% vs. 23.9% vs. 22.4%) and worked in second-class hospital (30.7% vs. 28.9% vs. 26.6% vs. 22.7% vs. 22.9%) and worked more than 20 years (5.5% vs. 6.8% vs. 9.4% vs. 14.0% vs. 20.5%) and had the title of ‘senior registered nurse’ (1.6% vs. 4.2% vs. 6.3% vs. 13.3% vs. 20.0%) and had the title of ‘senior registered nurse’ (1.6% vs. 5.8% vs. 4.7% vs. 15.4% vs. 21.9%) and were formal employed nurse (21.3% vs. 25.3% vs. 31.3% vs. 30.6% vs. 31.4%).

**Table 2:** Demographic and job-related features by latent profile membership

Variables	Overall (*N* = 1013) *n* (%)	Profile 1 (*n* = 210) *n* (%)	Profile 2 (*n* = 422) *n* (%)	Profile 3 (*n* = 64) *n* (%)	Profile 4 (*n* = 190) *n* (%)	Profile 5 (*n* = 127) *n* (%)
Gender						
Male	30(3.0)	7(3.3)	14(3.3)	3(4.7)	4(2.1)	2(1.6)
Female	983(97.0)	203(96.7)	408(96.7)	61(95.3)	186(97.9)	125(98.4)
Education level						
Associate degree and below	263(26.0)	47(22.4)	101(23.9)	15(23.5)	50(26.3)	50(39.4)
Bachelor’s degree	678(66.9)	146(69.5)	293(69.4)	42(65.6)	128(67.4)	69(54.3)
Master’s degree and above	72(7.1)	17(8.1)	28(6.7)	7(10.9)	12(6.3)	8(6.3)
Marital status						
Single	298(29.4)	53(25.2)	115(27.2)	21(32.8)	61(32.1)	48(37.8)
Married	685(67.6)	153(72.9)	294(69.7)	41(64.1)	124(65.3)	73(57.5)
Divorce	30(3.0)	4(1.9)	13(3.1)	2(3.1)	5(2.6)	6(4.7)
Have any children						
Yes	646(63.8)	147(70.0)	276(65.4)	38(59.4)	118(62.1)	67(52.8)
No	367(36.2)	63(30.0)	146(34.6)	26(40.6)	72(37.9)	60(47.2)
Hospital level						
Third-class	758(74.8)	162(77.1)	326(77.3)	47(73.4)	135(71.1)	88(69.3)
Second-class	255(25.2)	48(22.9)	96(22.7)	17(26.6)	55(28.9)	39(30.7)
Hospital department						
Internal Medicine	363(35.8)	74(35.2)	158(37.5)	23(35.9)	69(36.3)	39(30.7)
Surgery	170(16.8)	27(12.9)	86(20.4)	6(9.4)	32(16.9)	19(15.0)
Obstetrics and Gynecology	54(5.3)	24(11.4)	14(3.3)	3(4.7)	10(5.3)	3(2.4)
Pediatrics	49(4.8)	8(3.8)	17(4.0)	4(6.3)	12(6.3)	8(6.3)
Emergency treatment	94(9.3)	15(7.2)	39(9.2)	5(7.8)	19(10.0)	16(12.6)
ICU	62(6.1)	9(4.3)	26(6.2)	5(7.8)	11(5.8)	11(8.6)
Operating room	22(2.2)	7(3.3)	6(1.4)	5(7.8)	1(0.5)	3(2.4)
Other	199(19.7)	46(21.9)	76(18.0)	13(20.3)	36(18.9)	28(22.0)
Years of nursing experience						
< 2 years	130(12.8)	26(12.4)	51(12.1)	4(6.2)	28(14.7)	21(16.5)
2–5 years	188(18.6)	29(13.8)	76(18.0)	15(23.4)	33(17.4)	35(27.6)
6–10 years	235(23.2)	38(18.1)	94(22.3)	20(31.3)	53(27.9)	30(23.6)
11–20 years	332(32.8)	74(35.2)	142(33.6)	19(29.7)	63(33.2)	34(26.8)
>20 years	128(12.6)	43(20.5)	59(14.0)	6(9.4)	13(6.8)	7(5.5)
Professional title						
Junior Registered Nurse	193(19.1)	33(15.7)	73(17.3)	9(14.1)	44(23.1)	34(26.8)
Senior Registered Nurse	361(35.6)	56(26.7)	150(35.5)	31(48.4)	71(37.4)	53(41.7)
Supervisor nurse	347(34.3)	79(37.6)	143(33.9)	20(31.3)	67(35.3)	38(29.9)
Associate professor or professor nurses	112(11.1)	42(20.0)	56(13.3)	4(6.2)	8(4.2)	2(1.6)
Hospital position						
Nurse	692(68.3)	120(57.1)	280(66.3)	46(71.9)	146(76.8)	100(78.7)
Teaching nurses	83(8.2)	21(10.0)	32(7.6)	4(6.2)	14(7.4)	12(9.5)
Nursing team leader	111(11.0)	23(11.0)	45(10.7)	11(17.2)	19(10.0)	13(10.2)
Chief nurse	127(12.5)	46(21.9)	65(15.4)	3(4.7)	11(5.8)	2(1.6)
Employment type						
Formal employed nurse	290(28.6)	66(31.4)	129(30.6)	20(31.2)	48(25.3)	27(21.3)
Contract employed nurse	723(71.4)	144(68.6)	293(69.4)	44(68.8)	142(74.7)	100(78.7)

Profile 1: High prosocial job characteristics profile, Profile 2: Moderate prosocial job characteristics profile, Profile 3: High social worth-low social impact perceived profile, Profile 4: Low social worth‐high social impact perceived profile, Profile 5: Low prosocial job characteristics profile

### Predictor of latent profile membership

To identify the predictors of profile membership, a multinomial logistic regression was conducted with the ‘high prosocial job characteristics’ group as the reference. The Predictors are highlighted in bold in Table 3. Nurses worked in surgery were more likely to be in the ‘moderate prosocial job characteristics’ group compared with those in the ‘high prosocial job characteristics’ group (OR = 1.863, p = 0.042). The older nurses were more likely to be in the ‘high social worth-low social impact perceived’ group compared with those in the ‘high prosocial job characteristics’ group (OR = 1.863, p = 0.042). Nurses had the title of ‘junior registered nurse’ were more likely to be in the ‘low social worth‐high social impact perceived’ group compared with those in the ‘high prosocial job characteristics’ group (OR = 5.490, *p* = 0.028). Divorced nurses were more likely to be in the ‘low prosocial job characteristics’ group compared with those in the ‘high prosocial job characteristics’ group (OR = 0.145, *p* = 0.010). Compared to individuals in other group, nurses who worked in obstetrics and gynecology and worked more than 20 years and the chief nurse were more likely to be in the ‘high prosocial job characteristics’ group

**Table 3:** Predictor of latent profile membership

	B	SE	OR	95% confidence interval	*p*		B	SE	OR	95% confidence interval	*p*
**Profile 2: Moderate prosocial job characteristics profile (vs. Profile 1: High prosocial job characteristics profile)**						**Profile 3: High social worth-low social impact perceived profile (vs. Profile 1: High prosocial job characteristics profile)**					
Age, y: numerical value	0.014	0.030	1.014	0.955–1.076	0.653	**Age, y: numerical value**	**0.198**	**0.057**	**1.220**	**1.092–1.362**	**0.000**
Gender: male, ref.: female	−0.016	0.509	0.985	0.363–2.667	0.976	Gender: male, ref.: female	−0.337	0.803	0.714	0.148–3.442	0.674
Hospital level: third-class, ref.: second-class	0.018	0.229	1.018	0.650–1.595	0.938	Hospital level: third-class, ref.: second-class	−0.234	0.381	0.791	0.375–1.669	0.538
Hospital department: internal Medicine, ref.: other	0.168	0.249	1.183	0.726–1.926	0.500	Hospital department: internal Medicine, ref.: other	−0.050	0.433	0.952	0.407–2.224	0.909
**Hospital department: surgery, ref.: other**	**0.622**	**0.306**	**1.863**	**1.023–3.392**	**0.042**	Hospital department: surgery, ref.: other	−0.326	0.591	0.722	0.226-2.300	0.581
**Hospital department: obstetrics and gynecology, ref.: other**	**−1.101**	**0.405**	**0.333**	**0.150–0.736**	**0.007**	Hospital department: obstetrics and gynecology, ref.: other	−1.032	0.732	0.356	0.085–1.497	0.159
Hospital department: pediatrics, ref.: other	0.106	0.483	1.112	0.431–2.865	0.826	Hospital department: pediatrics, ref.: other	0.271	0.742	1.311	0.306–5.615	0.715
Hospital department: emergency treatment, ref.: other	0.355	0.387	1.426	0.668–3.043	0.359	Hospital department: emergency treatment, ref.: other	0.019	0.669	1.019	0.274–3.782	0.978
Hospital department: ICU, ref.: other	0.362	0.457	1.437	0.586–3.521	0.428	Hospital department: ICU, ref.: other	0.259	0.704	1.296	0.326–5.153	0.713
Hospital department: operating room, ref.: other	−0.814	0.613	0.443	0.133–1.473	0.184	Hospital department: operating room, ref.: other	0.865	0.738	2.374	0.559–10.087	0.241
Years of nursing experience: <2 years, ref.: >20 years	−0.038	0.761	0.963	0.217–4.279	0.960	Years of nursing experience: <2 years, ref.: >20 years	2.106	1.484	8.219	0.449-150.522	0.156
Years of nursing experience: 2–5 years, ref.: >20 years	0.236	0.667	1.266	0.343–4.674	0.724	**Years of nursing experience: 2–5 years, ref.: >20 years**	**3.279**	**1.288**	**26.541**	**2.125–331.486**	**0.011**
Years of nursing experience: 6–10 years, ref.: >20 years	0.275	0.538	1.317	0.458–3.782	0.609	**Years of nursing experience: 6–10 years, ref.: >20 years**	**3.094**	**1.073**	**22.066**	**2.691–180.915**	**0.004**
Years of nursing experience: 11–20 years, ref.: >20 years	0.295	0.406	1.343	0.606–2.976	0.468	**Years of nursing experience: 11–20 years, ref.: >20 years**	**2.107**	**0.837**	**8.223**	**1.593–42.451**	**0.012**
Professional title: junior registered nurse, ref.: associate professor or professor nurses	0.727	0.607	2.070	0.630–6.796	0.230	Professional title: junior registered nurse, ref.: associate professor or professor nurses	1.956	1.019	7.074	0.960–52.145	0.055
Professional title: senior registered nurse, ref.: associate professor or professor nurses	0.635	0.457	1.886	0.770–4.620	0.165	Professional title: senior registered nurse, ref.: associate professor or professor nurses	1.619	0.826	5.051	1.000–25.517	0.050
Professional title: supervisor nurse, ref.: associate professor or professor nurses	0.224	0.348	1.251	0.632–2.476	0.520	Professional title: supervisor nurse, ref.: associate professor or professor nurses	0.563	0.705	1.756	0.441–6.991	0.425
Hospital position: nurse, ref.: chief nurse	0.299	0.307	1.349	0.739–2.460	0.329	Hospital position: nurse, ref.: chief nurse	1.264	0.734	3.541	0.841–14.911	0.085
Hospital position: teaching nurse, ref.: chief nurse	0.049	0.388	1.051	0.491–2.248	0.899	Hospital position: teaching nurse, ref.: chief nurse	0.957	0.885	2.604	0.460–14.752	0.280
Hospital position: nursing team leader, ref.: chief nurse	0.099	0.357	1.104	0.548–2.222	0.782	**Hospital position: nursing team leader, ref.: chief nurse**	**1.895**	**0.770**	**6.653**	**1.471–30.100**	**0.014**
Employment type: formal employed nurse, ref.: contract employed nurse	0.378	0.230	1.459	0.929–2.291	0.101	Employment type: formal employed nurse, ref.: contract employed nurse	0.435	0.385	1.544	0.727–3.282	0.258
Education level: associate degree and below, ref.: master’s degree and above	0.065	0.417	1.067	0.471–2.418	0.876	Education level: associate degree and below, ref.: master’s degree and above	−0.699	0.663	0.497	0.136–1.822	0.292
Education level: bachelor’s degree, ref.: master’s degree and above	0.173	0.364	1.189	0.582–2.426	0.635	Education level: bachelor’s degree, ref.: master’s degree and above	−0.385	0.570	0.680	0.223–2.080	0.499
Marital status: single, ref.: divorce	−1.196	0.739	0.302	0.071–1.286	0.105	Marital status: single, ref.: divorce	−0.620	1.094	0.538	0.063–4.595	0.571
Marital status: married, ref.: divorce	−0.895	0.613	0.409	0.123–1.358	0.144	Marital status: married, ref.: divorce	−0.737	0.950	0.479	0.074–3.078	0.438
Have any children: yes, ref.: no	−0.285	0.401	0.752	0.343–1.652	0.478	Have any children: yes, ref.: no	−0.522	0.618	0.593	0.177–1.993	0.399
**Profile 4: Low social worth-high social impact perceived profile (vs. Profile 1: High prosocial job characteristics profile)**						**Profile 5: Low prosocial job characteristics profile (vs. Profile 1: High prosocial job characteristics profile)**					
Age, y: numerical value	−0.027	0.039	0.973	0.901–1.051	0.485	Age, y: numerical value	−0.072	0.048	0.931	0.846–1.023	0.138
Gender: male, ref.: female	−0.399	0.676	0.671	0.178–2.526	0.555	Gender: male, ref.: female	−1.342	0.855	0.261	0.049–1.396	0.117
Hospital level: third-class, ref.: second-class	−0.327	0.263	0.721	0.431–1.207	0.214	Hospital level: third-class, ref.: second-class	−0.316	0.297	0.729	0.407–1.306	0.288
Hospital department: internal Medicine, ref.: other	−0.125	0.302	0.883	0.489–1.594	0.679	Hospital department: internal Medicine, ref.: other	−0.586	0.342	0.557	0.285–1.089	0.087
Hospital department: surgery, ref.: other	0.200	0.371	1.221	0.590–2.528	0.591	Hospital department: surgery, ref.: other	−0.201	0.421	0.818	0.358–1.868	0.634
Hospital department: obstetrics and gynecology, ref.: other	−0.862	0.474	0.422	0.167–1.069	0.069	**Hospital department: obstetrics and gynecology, ref.: other**	**−1.935**	**0.700**	**0.144**	**0.037–0.570**	**0.006**
Hospital department: pediatrics, ref.: other	0.298	0.535	1.347	0.472–3.845	0.578	Hospital department: pediatrics, ref.: other	−0.090	0.596	0.913	0.284–2.937	0.879
Hospital department: emergency treatment, ref.: other	0.105	0.456	1.111	0.454–2.716	0.818	Hospital department: emergency treatment, ref.: other	0.082	0.493	1.086	0.413–2.855	0.868
Hospital department: ICU, ref.: other	0.049	0.542	1.050	0.363–3.035	0.928	Hospital department: ICU, ref.: other	0.088	0.565	1.092	0.361–3.307	0.876
Hospital department: operating room, ref.: other	−1.968	1.121	0.140	0.016–1.258	0.079	Hospital department: operating room, ref.: other	−0.359	0.783	0.698	0.151–3.239	0.647
Years of nursing experience: <2 years, ref.: >20 years	−0.858	0.956	0.424	0.065–2.759	0.369	Years of nursing experience: <2 years, ref.: >20 years	−0.867	1.175	0.420	0.042–4.207	0.461
Years of nursing experience: 2–5 years, ref.: >20 years	−0.578	0.853	0.561	0.106–2.985	0.498	Years of nursing experience: 2–5 years, ref.: >20 years	−0.247	1.047	0.781	0.100-6.078	0.813
Years of nursing experience: 6–10 years, ref.: >20 years	0.108	0.697	1.114	0.284–4.369	0.877	Years of nursing experience: 6–10 years, ref.: >20 years	−0.275	0.876	0.759	0.136–4.227	0.753
Years of nursing experience: 11–20 years, ref.: >20 years	0.184	0.559	1.202	0.402–3.598	0.742	Years of nursing experience: 11–20 years, ref.: >20 years	−0.110	0.718	0.896	0.219–3.661	0.878
**Professional title: junior registered nurse, ref.: associate professor or professor nurses**	**1.703**	**0.774**	**5.490**	**1.205–25.022**	**0.028**	Professional title: junior registered nurse, ref.: associate professor or professor nurses	1.307	1.050	3.693	0.471–28.942	0.214
Professional title: senior registered nurse, ref.: associate professor or professor nurses	1.155	0.629	3.173	0.925–10.881	0.066	Professional title: senior registered nurse, ref.: associate professor or professor nurses	1.261	0.924	3.528	0.577–21.579	0.172
Professional title: supervisor nurse, ref.: associate professor or professor nurses	0.851	0.531	2.341	0.826–6.631	0.109	Professional title: supervisor nurse, ref.: associate professor or professor nurses	1.044	0.843	2.840	0.544–14.826	0.216
**Hospital position: nurse, ref.: chief nurse**	**1.025**	**0.440**	**2.788**	**1.177–6.602**	**0.020**	**Hospital position: nurse, ref.: chief nurse**	**1.951**	**0.804**	**7.038**	**1.455–34.049**	**0.015**
Hospital position: teaching nurse, ref.: chief nurse	0.608	0.536	1.836	0.643–5.246	0.257	**Hospital position: teaching nurse, ref.: chief nurse**	**2.105**	**0.866**	**8.205**	**1.503–44.783**	**0.015**
Hospital position: nursing team leader, ref.: chief nurse	0.724	0.495	2.062	0.782–5.438	0.144	**Hospital position: nursing team leader, ref.: chief nurse**	**2.086**	**0.845**	**8.056**	**1.539–42.180**	**0.014**
Employment type: formal employed nurse, ref.: contract employed nurse	0.486	0.276	1.627	0.948–2.791	0.077	Employment type: formal employed nurse, ref.: contract employed nurse	0.418	0.323	1.518	0.806–2.861	0.196
Education level: associate degree and below, ref.: master’s degree and above	−0.191	0.506	0.826	0.306–2.228	0.706	Education level: associate degree and below, ref.: master’s degree and above	0.399	0.579	1.490	0.479–4.629	0.491
Education level: bachelor’s degree, ref.: master’s degree and above	0.057	0.450	1.059	0.439–2.555	0.899	Education level: bachelor’s degree, ref.: master’s degree and above	0.016	0.527	1.016	0.362–2.856	0.975
Marital status: single, ref.: divorce	−0.901	0.878	0.406	0.073–2.272	0.305	**Marital status: single, ref.: divorce**	**−2.199**	**0.890**	**0.111**	**0.019–0.634**	**0.013**
Marital status: married, ref.: divorce	−1.037	0.736	0.355	0.084–1.501	0.159	**Marital status: married, ref.: divorce**	**−1.930**	**0.752**	**0.145**	**0.033–0.634**	**0.010**
Have any children: yes, ref.: no	0.098	0.481	1.103	0.430–2.832	0.838	Have any children: yes, ref.: no	−0.279	0.491	0.757	0.289–1.980	0.570

### Turnover intention with latent profile membership

Analysis of variance was conducted to explore the differences in the turnover intention of the five profiles (Table 4). The mean scores of the turnover intention of nurses in Profiles 1, 2, 3, 4 and 5 were 13.44 (SD = 2.94), 14.03 (SD = 2.23), 14.47 (SD = 2.23), 14.95 (SD = 2.23) and 14.77 (SD = 2.27), respectively. As shown in Table 4, the scores of the turnover intention and the three dimensions statistically differed across the five profiles (*p* < 0.001). Moreover, the LSD test revealed that the mean score of the ‘high prosocial job characteristics’ group was significantly lower than other groups

**Table 4:** Turnover intention difference of five profiles

	Profile 1 (*n* = 210) M ± SD	Profile 2 (*n* = 422) M ± SD	Profile 3 (*n* = 64) M ± SD	Profile 4 (*n* = 190) M ± SD	Profile 5 (*n* = 127) M ± SD	F	*p*	LSD
Turnover Intention	13.44 ± 2.94	14.03 ± 2.23	14.47 ± 2.23	14.95 ± 2.23	14.77 ± 2.27	12.529	0.000	1 < 2 < 4 1 < 2 < 5 1 < 3
The possibility of quitting your current job	3.76 ± 1.77	4.52 ± 1.55	5.08 ± 1.66	5.18 ± 1.59	5.08 ± 1.50	24.932	0.000	1 < 2 < 3 1 < 2 < 4 1 < 2 < 5
Motivation to find other jobs	4.20 ± 1.33	4.59 ± 1.03	4.78 ± 1.05	5.04 ± 1.02	5.08 ± 1.09	19.989	0.000	1 < 2 < 4 1 < 2 < 5 1 < 3
The possibility of getting an outside job	5.48 ± 1.29	4.92 ± 1.14	4.61 ± 1.29	4.73 ± 0.97	4.61 ± 1.22	16.492	0.000	1 < 2 < 3 1 < 2 < 5 1 < 4

### Subjective well-being with latent profile membership

Analysis of variance was conducted to explore the differences in the subjective well-being of the five profiles (Table 5). The mean scores of the turnover intention of nurses in Profiles 1, 2, 3, 4 and 5 were 11.05 (SD = 1.51), 9.66 (SD = 1.58), 8.95 (SD = 1.93), 8.50 (SD = 1.74) and 7.87 (SD = 1.72), respectively. As shown in Table 5, the scores of the turnover intention and the three dimensions statistically differed across the five profiles (*p* < 0.001). Moreover, the LSD test revealed that the mean score of the ‘high prosocial job characteristics’ group was significantly higher than other groups, and the figure for the ‘low prosocial job characteristics’ group was the lowest

**Table 5:** Subjective well-being difference of five profiles

	Profile 1 (*n* = 210) M ± SD	Profile 2 (*n* = 422) M ± SD	Profile 3 (*n* = 64) M ± SD	Profile 4 (*n* = 190) M ± SD	Profile 5 (*n* = 127) M ± SD	F	*p*	LSD
Subjective well-being	11.05 ± 1.51	9.66 ± 1.58	8.95 ± 1.93	8.50 ± 1.74	7.87 ± 1.72	99.637	0.000	1 > 2 > 3 > 5 1 > 2 > 4 > 5
General affect	6.17 ± 1.09	5.42 ± 1.23	4.98 ± 1.26	4.64 ± 1.30	4.30 ± 1.38	62.136	0.000	1 > 2 > 3 > 5 1 > 2 > 4 > 5
Life satisfaction	4.44 ± 0.67	3.86 ± 0.62	3.61 ± 0.81	3.51 ± 0.75	3.24 ± 0.65	81.452	0.000	1 > 2 > 3 > 5 1 > 2 > 4 > 5

## Discussion

### Latent profiles of relational job characteristics

Latent profile analysis can identify heterogeneity between individual clinical nurses. This study used a person-centered approach to analyze the relational job characteristics of nurses with the aim of highlighting differences in their relational job characteristics and guiding further targeted research on improving relational job characteristics based on latent profiles. Meanwhile, as the first study to apply latent profile analysis to the relational job characteristics of clinical nurses, this study complements previous studies that have viewed nurses as a homogeneous whole. Thus, this study contributes to the development of targeted interventions based on the different profiles of nurses’ characteristics

The findings of this study revealed the distinct categorical features of the relational job characteristics among nurses. Based on the score responses for each item, five profiles were identified, namely, the ‘high prosocial job characteristics’, ‘moderate prosocial job characteristics’, ‘high social worth-low social impact perceived’, ‘low social worth‐high social impact perceived’ and ‘low prosocial job characteristics’ groups. This classification reflects the heterogeneity of nurses in each latent profile and can be used as a reference for comparison in the future

The ‘high prosocial job characteristics’ group consisted of 20.7% of the sample. Nurses in this subgroup had the highest scores of all items amongst the five subgroups. This result indicates that nurses in this subgroup have the best social worth perception and social impact perception, that is, they are more likely to produce prosocial behaviors at work. Some results show that under the influence of nursing education, hospital management, social cognition and related public opinion, nurses are generally regarded as a profession that requires more responsibility and humanistic care ability [37–39]. Therefore, under the influence of relevant factors, this subgroup of nurses may be more likely to recognize the social worth and social impact brought by the nursing profession, and thus show the high prosocial job characteristics

The ‘moderate prosocial job characteristics’ group has the highest proportion of 41.7%. The average score of the relational job characteristics of nurses in this subgroup is 5.9, which is the group closest to the overall level of clinical nurses [22]. Although education and public opinion require nurses to have prosocial behavior at work, in real life, busy work, huge pressure and frequent doctor-patient disputes will inevitably consume nurses’ work enthusiasm [40]. Therefore, under the combined influence of education and work pressure, this subgroup of nurses may develop moderate prosocial job behavior. These results indicate that most clinical nurses need to receive professional training related to prosocial behavior to resist some of the negative effects of their work in order to provide better nursing services for patients

The ‘high social worth-low social impact perceived’ group, comprising 6.3%, had relatively low scores on dimension perceived social impact. This subgroup of nurses had a higher recognition of the social worth of nursing work, but a lower recognition of the social impact of their work. This may be due to the low self-confidence of this subgroup of nurses in their own ability to work [41]. As a result, although they recognized the social worth of nursing work, they could not well affirm the positive impact of their work on the society. Therefore, the nurses in this subgroup should be trained to improve their ability and self-confidence in their work, so that they can understand the positive impact of their work on the patients and the society, so as to improve their relational job characteristics

The ‘low social worth-high social impact perceived’ group, comprising 18.8%, had relatively low scores on dimension perceived social worth. This subgroup of nurses had low overall scores on relational job characteristics and recognized the social worth of nursing less than the social impact of their work. This result may indicate that social worth perception has a more important impact on improving nurses’ relational job characteristics. Therefore, in the face of this subgroup of nurses, we need to increase the promotion of the worth perception of nursing work on the basis of prosocial job behavior training. In order to increase the identity and sense of honor of nursing work in this subgroup of nurses, which will improve their relational job characteristics

The ‘low prosocial job characteristics’ group, which accounted for the remaining 12.5% of the sample, had the lowest level of relational job characteristics items. Their low prosocial job characteristics can be attributed to several factors. First, high work pressure. By the end of 2022, the number of nurses in China reached 5.22 million, with about 3.71 registered nurses per 1,000 people [42], just reaching the global level of 3.69 nurses per 1,000 people in 2018 [43]. From this, we can see that China’s human resources for nurses are still tight. In addition, nursing work has high pressure, high demand and high intensity work requirements in terms of interpersonal interaction with different groups and responsibility for patients. As a result, nurses are faced with high occupational pressure, which may reduce their work enthusiasm, resulting in low prosocial job behavior. Secondly, previous studies have found that the professional identity of Chinese nurses is mostly at a medium level and needs to be further improved [44]. Professional identity is the inner motivation to achieve career goals, and high professional identity can stimulate the enthusiasm of individuals in work [16]. Therefore, the lack of professional identity may also be an important reason for the low prosocial job characteristics of nurses. Finally, frequent doctor-patient disputes cause nurses to worry about health threats from patients in addition to work pressure, which will inevitably shake nurses’ positive cognition of the worth and impact of their work, thus reducing their relational job characteristics

### Demographic and job-related characteristics of each profile

Demographic predictors of profile membership include age and marital status. Compared with the ‘high prosocial job characteristics’ group, high-age nurses were more likely to enter the ‘high social worth-low social impact perceived’ group, and divorced nurses were more likely to be in the ‘low prosocial job characteristics’ group. This may be due to the fact that older nurses, on the one hand, have long received value publicity from hospitals and leaders in their work, and have a higher recognition of the worth of nursing work [45]. At the same time, they also witnessed a lot of patient deaths, which inevitably reduced their confidence in the positive impact of nursing work on patients and society [46]. Therefore, nursing management or policy makers should pay more attention to the perceptions of elderly nurses about the social impact of nursing and provide timely psychological interventions. The divorced nurses may be affected by the negative effects of their own living conditions, such as loneliness and depression, which affect their work status [47]. It is suggested that nursing management or policy makers should pay more attention to the psychological status of divorced nurses, and carry out corresponding psychological intervention when necessary, so as to improve their mental health level and relational job characteristics

The job-related predictors of profile membership in this study include hospital department, years of nursing experience, professional title and hospital position. Compared with the ‘high prosocial job characteristics’ group, surgical nurses were more likely to enter the ‘moderate prosocial job characteristics’ group, nurses with less than 20 years of service were more likely to enter the ‘high social worth-low social impact perceived’ group than nurses with more than 20 years of service, and clinical nurses with the title of ‘nurse’ were more likely to enter the ‘low social worth‐high social impact perceived’ group. This may be due to the fact that surgical nurses are exposed to more bloody scenes on a daily basis, which affects their mental health, thereby reducing their positive evaluation of nursing work and leading to a decrease in their prosocial job behavior [48]. Nurses with less than 20 years of service may, on the one hand, not be able to get rid of the feeling of helplessness in front of critically ill patients due to the lack of life experience. On the other hand, they may lack self-confidence in their ability to care for patients due to their lack of nursing experience, which reduces their perception of social impact [49]. Clinical nurses with the title of ‘nurse’ may be in the adaptation stage of changing their status from nursing students to nurses because they have just entered the clinic, and maladaptation at work may have contributed to their low worth perception of nursing work [50]. This suggests that nursing education or managers need to pay more attention to the relational job characteristics of clinical nurses in surgery, with less than 20 years of service, and with the title of nurse, and to target psychological interventions and prosocial behavioral development for them

Obstetrics and gynecology nurses, those with more than 20 years of service and chief nurse are more likely to belong to the ‘high prosocial job characteristics’ group compared to other profiles. This may be due to the fact that obstetrics and gynecology nurses are exposed to more newborn babies and the joy of a new birth creates a good working atmosphere, which in turn produces a high prosocial job behavior [51]. Working experience of more than 20 years means that these nurses have sufficient experience, have established stable interpersonal relationships with their colleagues and have developed a strong sense of attachment to the hospital and their work environment, thus being more likely to develop high relational job characteristics. Chief nurse are the grassroots managers and organizers of the hospital nursing team. The position proves that they have high nursing ability, and prosocial job ability is included in it

Therefore, good working environment, stable interpersonal relationships and excellent nursing work ability may be the key to enhancing nurses’ relational job characteristics. In the future, nursing education and managers can formulate intervention programs for nursing relational job characteristics from these aspects

### Turnover intention and subjective well-being of the five profiles

The total score and dimensions of turnover intention of the ‘high prosocial job characteristics’ group was notably lower than those of the other four groups, and the average score of life satisfaction, general affect and subjective well-being of the ‘high prosocial job characteristics’ group was notably higher than those of the other four groups. This indicates that the nurses in the ‘high prosocial job characteristics’ group have higher subjective well-being and lower turnover intention, similar to the results of previous studies [19, 22]. Therefore, the ‘high prosocial job characteristics’ group is the most suitable type of nurses for clinical work. This suggests that nursing education and administrators should focus on the relational job characteristics of nurses and develop appropriate prosocial job ability training to help clinical nurses better recognize the positive social worth and social impacts of nursing work, thereby reducing their turnover intention and increasing their prosocial job behavior and subjective well-being. To help hospitals and patients gain a more stable, active nursing team and higher quality nursing behavior

## Limitations

There are several limitations of this study that should be acknowledged. Firstly, participants in this study were recruited via snowball sampling. This sampling method may introduce selection bias and decrease the representativeness of the study sample. Future studies should utilize random sampling to improve the representativeness of the study sample. Secondly, the study is a cross-sectional study and identification of causal relationships between variables may not be possible. Therefore, further longitudinal studies are recommended to follow up the trajectory of relational job characteristics amongst nurses. Thirdly, since most of the participants were female nurses, gender bias may not be completely avoided. Future studies should recruit more male nurses. Finally, the nurses were recruited from a single region of Asia, thus limiting the representativeness and generalization of the sample

## Conclusions

The nurses in the ‘high prosocial job characteristics’ group had the lowest total score of turnover intention and three dimensions, and the highest total score of subjective well-being and two dimensions. Therefore, the nurses in the ‘high prosocial job characteristics’ group are the most suitable nurses for clinical work. This suggests that nursing education and management should carry out relevant training in time to improve the relational job characteristics of clinical nurses. When developing targeted interventions for nurses’ relational job characteristics, nursing educators and administrators should pay attention to the characteristics of each profile, as shown in the LPA results. For the ‘moderate prosocial job characteristics’ group and ‘low prosocial job characteristics’ group, a balanced mix of perceived social worth and perceived social impact interventions can be implemented through improving the departmental atmosphere, solidifying interpersonal relationships, and enhancing skills training. For the ‘low social worth-high social impact perceived’ group, we need to focus on training nurses’ social worth cognition, through case lectures, banners and other methods to strengthen their cognition of the worth and significance of nursing work. For the ‘high social worth‐low social impact perceived’ group, attention should be paid to strengthening the social impact perception of this subgroup of nurses, through health visits and other methods, to help this subgroup of nurses to understand the positive impact they have brought to the patients and the community, so as to improve their relational job characteristics

## Data Availability

The datasets used and analysed during the current study are available from the corresponding author on reasonable request.
